# Antioxidant and Antibacterial Potential of Silver Nanoparticles: Biogenic Synthesis Utilizing Apple Extract

**DOI:** 10.1155/2016/7141523

**Published:** 2016-11-28

**Authors:** Upendra Nagaich, Neha Gulati, Swati Chauhan

**Affiliations:** Department of Pharmaceutics, Amity Institute of Pharmacy, Amity University, Noida, Uttar Pradesh, India

## Abstract

The advancement of the biological production of nanoparticles using herbal extracts performs a significant role in nanotechnology discipline as it is green and does not engage harsh chemicals. The objective of the present investigation was to extract flavonoids in the mode of apple extract and synthesize its silver nanoparticles and ultimately nanoparticles loading into hydrogels. The presence of flavonoids in apple extract was characterized by preliminary testing like dil. ammonia test and confirmatory test by magnesium ribbon test. The synthesized silver nanoparticles were characterized using UV spectroscopy, particle size and surface morphology, and zeta potential. Silver nanoparticles loaded hydrogels were evaluated for physical appearance, pH, viscosity, spreadability, porosity,* in vitro* release,* ex vivo* permeation, and antibacterial (*E. coli* and* S. aureus*) and antioxidant studies (DPPH radical scavenging assay). Well dispersed silver nanoparticles below were observed in scanning electron microscope image. Hydrogels displayed* in vitro* release of 98.01%  ±  0.37% up to 24 h and* ex vivo* permeation of 98.81  ±  0.24% up to 24 h. Hydrogel effectively inhibited the growth of both microorganism indicating good antibacterial properties. The value of percent radical inhibition was 75.16%  ±  0.04 revealing its high antioxidant properties. As an outcome, it can be concluded that antioxidant and antiageing traits of flavonoids in apple extract plus biocidal feature of silver nanoparticles can be synergistically and successfully utilized in the form of hydrogel.

## 1. Introduction

 Nanotechnology is an emerging tool as drug delivery system in variety of serious disorders. It is an innovative technique which includes the design, characterization, production, and application of structures, devices, and systems by controlling shape and size at the nanometer scale. It covers the size range of 1 nm to 100 nm [1]. Nanoparticles are the materials with the overall size of 100 nm [2]. Nanoparticles can be classified as polymeric (natural and synthetic), lipoidal (biodegradable), and metal nanoparticles (iron oxide, gold nanoparticles, and silver nanoparticles) [3]. Metal nanoparticles using plant or plant extract are a way ahead towards greener approach for application as drug delivery system. Silver nanoparticles show good anti bacterial properties as silver has been widely used as healing and antibacterial agent for many years [4]. Silver nanoparticles (AgNPs) also possess antifungal, anti-inflammatory, antiviral, and antiplatelet activity [5]. Silver nanoparticles can be synthesized by using any of the following methods [6], namely, physical methods, chemical reduction (thermal reduction, polyol process, microemulsion techniques), and photochemical reduction (microwave reduction, photoreduction, and X-ray radiolysis).

*Malus domestica* (Rosaceae) commonly known as apple is full of high levels of triterpenoids, anthocyanins, and phenolic compounds. Apple polyphenols and oligomeric proanthocyanidins are primarily responsible for its antioxidant activity [7]. Flavonoids, quercetin, and hesperetin present in apple contribute to its antiageing effects [8]. The utilization of natural compounds in protection of skin especially topical application of antioxidants indicates that they usefully decrease skin aging [9, 10].

Basically, premature skin ageing is a vast biological phenomenon that mainly occur by the combination of endogenous or intrinsic (genetics, cellular metabolism, hormone, and metabolic processes) and exogenous or extrinsic (chronic light exposure, pollution, ionizing radiation, chemicals, and toxins) factors [11]. Photosensitized skin is characterized by dry, rough, pigmented, and abraded skin. This is due to high exposure to direct sunlight [8]. Biological systems are damaged by the presence of free radicals. The responsible free radicals are oxygen free radicals or generally known as reactive oxygen species (ROS) [12].

Studies show that apple decreases the presence of ROS generated by hydrogen peroxide exposure in lymphocytes [13].

In present investigation, we have tried to investigate the synergistic effect of AgNPs and flavonoids in apple extract by loading apple extract AgNPs into hydrogel and characterizing it for antibacterial and antioxidant properties in a unit dose formulation. Apple extract was utilized for the green synthesis of silver nanoparticles via chemical reduction technique. Furthermore Apple extract acts as both reducing and capping agent. Moreover, optimized silver nanoparticles were selected and loaded to hydrogel to deliver actives via topical route for the therapy of premature skin ageing.

## 2. Materials and Methods

### 2.1. Materials

*Malus domestica*, family Rosaceae (apple), was purchased from local market. Methanol, formic acid, silver nitrate, carbopol-934, triethanolamine, glycerine, potassium dihydrogen phosphate, sodium hydroxide, 2,2-diphenyl-2-picrylhydrazyl hydrate (DPPH), agar, and nutrient medium were purchased from CDH Pvt. Ltd., New Delhi. All solvents used were of analytical grade.

### 2.2. Methodology

#### 2.2.1. Preparation of Apple Extract

Phenolic compounds from apple were extracted using solvent mixture of methanol : formic acid : water in the ratio of 70 : 2 : 28. Weighed quantity of freshly cut apple pieces was added to the solution of methanol : formic acid : water (MFW) and homogenized in a blender (Polytron PT 1600E, kinematica AG) for 2 min. The mixture was transferred to a beaker, covered with parafilm, and held for 24 h at 4°C. Then the mixture was washed with 20 mL of MFW. Extract was collected and then evaporated for dryness at 45°C under vacuum with a rotary evaporator [14].

#### 2.2.2. Synthesis of Silver Nanoparticles

1 M silver nitrate solution was prepared by adding 1.699 g of AgNO3 to 1 L distilled water. 30 mL of apple extract was taken in a 100 mL conical flask. 20 mL of freshly prepared 0.1 M AgNO3 solution was added (drop-wise) to the flask. The flask was incubated in dark for 24 hrs at room temperature [15].

#### 2.2.3. Preparation of Apple Extract Loaded Silver Nanoparticles Based Hydrogel

Apple extract loaded silver nanoparticles were then incorporated into a hydrogel base as given in Table 1. Accurately weighed quantity of carbopol-934 (poly (acrylic) acid or carbomer) was gradually dispersed in distilled water under mild stirring (Remi, Mumbai) for 30 min and kept for two hours for the proper swelling of polymer. Glycerin was then added as viscosity enhancer and emollient. The glycerin-polymeric solution was then neutralized with triethanolamine to form a transparent gel as basic nature of triethanolamine helps in increasing the cross-linking of acidic polymer. Apple extract loaded silver nanoparticulate suspension (equivalent to 3–10% of apple extract) was then incorporated in hydrogel using slow mechanical mixing (Remi RQ121/D, Mumbai) for 10 min so as to avoid entry of air bubbles.

#### 2.2.4. Characterization of Apple Extract Loaded Silver Nanoparticles

*(1) Characterization of Apple Extract*

*Preliminary Screening for the Presence of Flavonoids and Polyphenols in Extract*. The prepared apple extract was subjected to ultravoilet-visible spectrophotometry (Perkin Elmer, Waltham, MA) for detecting the presence of flavonoids. The extract was scanned in the region of 200 nm−800 nm against solvent mixture (MFW) as blank and peaks were obtained [16].

Few drops of dilute sodium hydroxide were added to 1 mL of apple extract to observe the yellow color which on further addition of few drops of dilute hydrochloric acid makes the solution colorless which confirms the presence of flavonoids.

*Confirmatory Testing (Magnesium Ribbon Test) [17]*. Magnesium ribbon test is known because of the use of magnesium ribbon in the testing of flavonoids. Concentrated hydrochloric acid and magnesium ribbon were added to 1 mL of apple extract which in turn gives pink-red color for the presence of flavonoids. 

*(2) Characterization of Apple Extract Loaded Silver Nanoparticles*

*Ultravoilet-Visible Spectrophotometry*. The optical property of prepared AgNPs was analyzed via UV-visible absorption spectrophotometer with a deuterium and tungsten iodine lamp in range of 200–800 nm at room temperature (RT). UV-visible spectrophotometer monitors the formation of AgNPs with the color change. To analyze formation of AgNPs, samples were withdrawn with the help of pipette at different time intervals, that is, 5, 10, 15, 20, 30, and 60 minutes [18].

*Shape and Surface Morphology of Silver Nanoparticles*. Scanning electron microscopic (SEM) analysis was done for analyzing particle shape and morphology. The shape and surface morphology of the silver nanoparticles was visualized by scanning electron microscopy (Cart Zeiss EV018). The samples were prepared by lightly sprinkling nanoparticles on double-sided adhesive tape on an aluminum stub. The stubs were then coated with gold to a thickness of 200 to 500 Å under an argon atmosphere using a gold sputter module in a high vacuum evaporator. The samples were then randomly scanned and photomicrographs were taken at different magnifications with SEM [19].

*Particle Size and Zeta Potential Measurement*. Particle size was measured with the help of dynamic light scattering technique. For the determination of particle size, samples were prepared by tenfold dilution of 1 mL of the nanoparticulate suspension with distilled water. The analysis was carried out in triplicate. The average particle size was measured by photon correlation spectroscopy. Zeta potential was determined by the electrophoretic mobility of nanoparticles in U-type tube at 25°C, using a zetasizer (3000HS Malvern Instruments, UK) [19].

*(3) Characterization of Apple Extract Loaded Silver Nanoparticles Based Hydrogels*

*pH*. pH of prepared hydrogel was measured by dissolving 1 gm of hydrogel in 10 mL of distilled water. pH meter (Systronics, 361-micro pH meter) was used to evaluate the pH of hydrogel. The readings were taken in triplicate and the average pH was calculated.

*Porosity Measurement*. For porosity measurement, the solvent replacement technique was employed. Accurately weighed 1 gm of dried hydrogels was immersed in absolute ethanol and soaked overnight. Afterwards, excess ethanol on the surface of hydrogels was blotted and weighed again [20]. The porosity can be calculated from the following equation.

(1) $$\begin{array}{c} \text{Porosity}=\frac{\left(M2-M1\right)}{\rho V}. \end{array}$$

Here, *M*1 and *M*2 are the mass of hydrogel before and after the immersion in absolute ethanol, respectively; *ρ* is the density of absolute ethanol and *V* is the volume of the hydrogel.

*Viscosity*. To analyze the rheological properties, all the formulated gels were taken in beakers and placed beneath the spindle, and the spindle (RV-7) was rotated at 10 rpm at room temperature (25–27°C) in Brookfield viscometer.

*Spreadability*. The Spreadability measurements of the apple extract loaded silver nanoparticle gel were made in triplicate by using glass slide method. One gram per meter of gel was kept between the two slides. The preweighted plate was kept above the gel, and more weights were added on the plate until the gel stop spreading. Final cumulative weight and the total time taken by the gel to spread were measured and noted. Then total weight applied and mass of the gel were compared by the time.

(2) $$\begin{array}{c} S=M\times \frac{L}{T}, \end{array}$$

where *S* = spreadability, *M* = weight tide to upper slide, *L* = length of glass slide, and *T* = time taken to separate the slides completely from each other.

#### 2.2.5. *In Vitro* Drug Diffusion Studies

The* in vitro* drug release was evaluated using Keshary-Chien (K-C) diffusion cell to evaluate the diffusion of nanoparticles across the dialysis membrane. The diffusion cells were thermoregulated with a water jacket at 37 ± 2°C. Dialysis membrane 70 (Hi-Media, Mumbai, India) having a pore size of 2.4 nm and a molecular weight cutoff between 12,000 and 14,000 Da was used and mounted on K-C cells. The surface area of the release membrane was 3.14 cm^2^. Phosphate buffer saline (PBS; pH 6.8) was used as the receptor medium (10 mL) being stirred at 100 rpm. 0.5 gm of apple extract loaded silver nanoparticulate based hydrogel (equivalent to 3–10% of apple extract) was placed in the donor compartment. During the experiments, the solution in receptor side was maintained at 37 ± 0.5°C. At predetermined time intervals, 5 mL of the samples was withdrawn from the receiver compartment and replaced by the same volume of freshly prepared PBS (pH 6.8). The withdrawn samples were analyzed by the UV-visible spectrophotometer at 420 nm [21].

#### 2.2.6. *Ex Vivo *Skin Permeation Studies

The* ex vivo* drug release studies were carried out with the help of K-C diffusion cell. Rat abdominal skin was used to study permeation. The subcutaneous tissue was removed surgically and the dermis side was wiped with isopropyl alcohol to remove adhering fat. The cleaned skin was washed with distilled water and stored at −18°C until further use. The skin was mounted between the donor and receiver compartments of the K-C cell where the stratum corneum side was facing the donor compartment and the dermal side was facing the receiver compartment. One gram (equivalent to 3–10% apple extract) of each gel was placed in a donor compartment. The receptor compartment was filled with 10 mL PBS (pH 6.8), thermoregulated at 37°C, and magnetically stirred at 400 rpm. Two milliliters of receptor fluid was withdrawn at an interval of 1, 2, 3, 5, 7, 12, 18, and 24 h. An equal volume of PBS was simultaneously added to the receptor compartment after each sampling to maintain sink conditions. Each sample was filtered and then determined for apple extract content by UV spectrophotometer at 420 nm. The concentrations of all the formulations in withdrawn samples were calculated and then the percent drug release was determined [22].

#### 2.2.7. Antioxidant Activity of Silver Nanoparticulate Hydrogel (DPPH Radical Scavenging Assay)

The antioxidant activity was characterized utilizing DPPH (2,2-diphenyl-2-picrylhydrazyl hydrate) assay. A stock solution of DPPH in methanol was prepared. 1 mL of this stock solution was added to 3 mL of hydrogel solution (1 gm of prepared hydrogel in 10 mL of distilled water). The mixture was shaken vigorously and allowed to stand at room temperature for 30 min. Then the absorbance was measured at 517 nm by using a UV-visible spectrophotometer. Antioxidant activity was estimated by calculating the % inhibition by following formula [23]:

(3) DPPH  scavenging  effect%=control  absorbance−sample  absorbancecontrol  absorbance×100.

#### 2.2.8. Antimicrobial Activity of Silver Nanoparticulate Hydrogel

Antimicrobial activity was performed using well-diffusion technique employing agar and nutrient broth as media. Media was constituted by mixing agar and nutrient broth and then autoclaved at 121°C at 15 lbsi for 60 min for sterilization. Sterilized liquid media were then poured into two different petri plates and inoculated with* E. coli *and* S. aureus,* respectively. The petri plates were kept for 5 min till solidification is complete. The entire work was carried out in laminar air flow unit. Sterilized cork borer was used to create well of 1 cm diameter in the solidified media. Samples were poured in the well of prepared petri plates and then incubated at 37 ± 5°C for 24 h for inhibition to the growth of* E. coli* and* S. aureus* [24].

## 3. Results and Discussion

Apple extract was successfully prepared using MFW solution as explained in literature [14]. Extract obtained by the process was found to be pinkish red in color; the extract was further screened for the presence of flavonoids and phenols by preliminary screening and confirmatory testing. The phytochemical analysis of apple extract confirms the presence of phenols and flavonoids.

**Figure 1:**
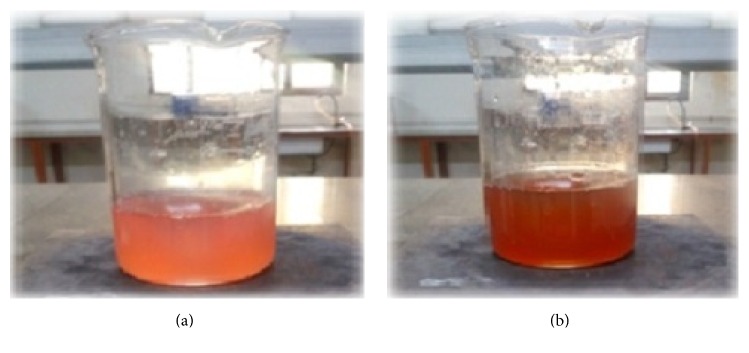
Formation of silver nanoparticles. (a) Color before initiation of reduction of silver ions; (b) color change after reduction.

Silver nanoparticles prepared from apple extract showed the color change after 120 minutes of preparation giving the evidence for the reduction of silver ions and, eventually, formation of silver nanoparticles. The color change of the solution is clearly depicted in Figures 1(a) and 1(b).

**Figure 2:**
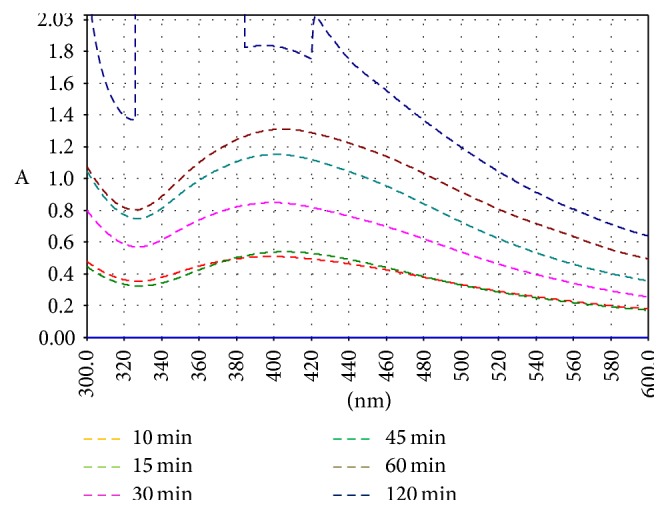
UV-visible spectra for apple extract loaded silver nanoparticles.

The color change was recorded by visual observation in beaker which contains silver nitrate solution with apple extract. The color of AgNPS apple extract solution changes from colorless to light brown after 5 min and eventually to dark brown as shown in Figures 1(a) and 1(b). This color change indicates formation of AgNPs in solution [25, 26]. The synthesis of AgNPs was furthermore confirmed by UV-visible spectroscopy and scanning electron microscopy [24]. The UV-Vis spectrum of silver nanoparticles was recorded as a function of a reaction time. Aliquots of samples were withdrawn at different time intervals, that is, 10, 15, 30, 45, 60, and 120 min, to monitor the bioreduction of Ag^+^ ions. The graph obtained by UV spectrophotometer contains significant bell shaped peak indicating the formation of silver nanoparticles as shown in Figure 2.

**Figure 3:**
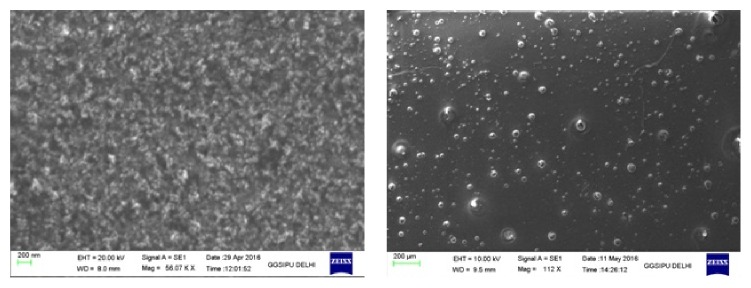
Scanning electron microscopic images of apple extract loaded silver nanoparticles.

Figure 2 shows UV-visible absorption spectrum of synthesized AgNPs. AgNPs have free electrons which give surface plasmon resonance (SPR) absorption band. This SPR absorption band may be attributed to combined variation of electrons of AgNPs in resonance with light wave. It is very much familiar that silver nanoparticles in nanorange show absorption at the wavelength from 390 nm to 420 nm due to Mie scattering. Therefore, a broad absorption band was observed in the range of 400 nm–440 nm which is the characteristic of Mie scattering [27, 28]. No other peak was recorded in spectrum which confirms that synthesized nanoparticles are silver particles only. SEM technique was employed to evaluate AgNPs size, shape, and surface morphology. The average size of the particles was found to be below 200 nm and the particles formed were spherical in shape, as shown in Figure 3.

From Figure 3, it can be illustrated that morphology of AgNPs is spherical and particles are well dispersed without any aggregation. The particle size of the silver nanoparticles was found to be in the range of 100 nm indicating best suitability for topical delivery as particles on nanoscale can easily permeate through skin. The zeta potential was found to be very negative on higher scale revealing good stability of AgNps in dispersion form. The prepared hydrogel was evaluated for pH, viscosity, spreadability, and porosity. The pH of all formulated hydrogel was found in the range of 5.5–7.5, which mimics the pH of skin; therefore, formulation shows no sign of irritation on skin. The results of rheological studies, namely, viscosity and spreadability of the formulation, were found to be 42000 cps and 5.56 ± 0.15 g·cm/sec, respectively, which clearly signify that the hydrogel is easy to spread on skin. The results of all the three batches formulated are mentioned in Table 2. Furthermore, hydrogel formulations were then subject to* in vitro* drug release to compute the rate and extent of drug release.

### 3.1. *In-Vitro* Release

**Figure 4:**
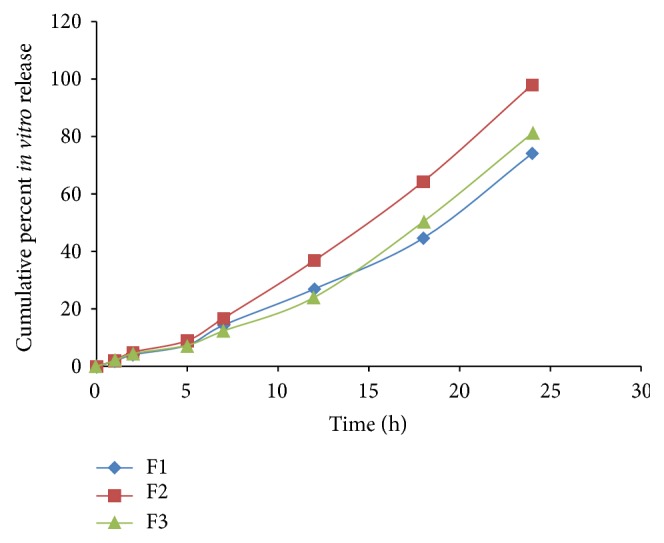
Cumulative percent* in vitro* release of apple extract loaded silver nanoparticles.

The results of* in vitro* drug release of all three formulations were in the range of 74.1%  ±  0.28 to 98.01%  ±  0.37 up to 24 h concluding the maximum release was shown by F2 hydrogel formulation as shown in Figure 4. On the basis of results in Table 2, optimized hydrogel formulation was selected. Primary focus was laid on particle size as this will play significant role in nanoparticle permeation from skin. The smaller the particle size is, the easier the permeation will be. Additionally, to confirm the permeation of nanoparticle across the membrane, rat abdominal skin was selected. F2 hydrogel formulation displayed the maximum release.

### 3.2. *Ex Vivo* Permeation Studies and Release Kinetics

**Figure 5:**
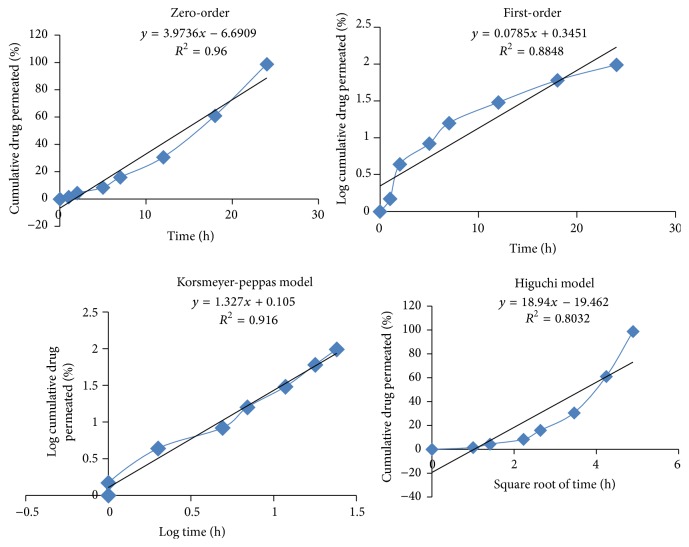
*Ex vivo* drug permeation kinetics of apple extract loaded silver nanoparticles based hydrogels.

*Ex vivo* permeation studies were only conducted for batch F2 as smaller particle size and maximum* in vitro* release was shown by this formulation. Cumulative percent permeation was found to be 98.81%  ±  0.24 up to 24 h. The permeation studies were best explained by zero-order kinetics (*R* ^2^ = 0.96), followed by first-order kinetics (*R* ^2^ = 0.884) and Higuchi equation (*R* ^2^ = 0.803).* Ex vivo* permeation kinetics through zero-order, first-order, Korsmeyer-Peppas model, and Higuchi model have been explained in Figure 5. The values of *R* ^2^ and *k* for all release kinetics models are tabulated in Table 3. Zero-order process is a constant rate process that is independent of drug concentration [29]. This perfectly highlights apple extract silver nanoparticles loaded hydrogels as sustained release dosage form. Permeation parameters like flux, permeability coefficient, and lag time are summarized in Table 3.

### 3.3. Antioxidant Activity of Apple Extract Loaded Silver Nanoparticles Loaded Hydrogels

DPPH radical scavenging assay was investigated for the evaluation of antioxidant potential of the prepared hydrogel formulations. The purple solution containing DPPH turns yellow on addition of formulation, which indicates the scavenging of free radicals and presence of antioxidant activity [30]. The results are compiled in Table 4 and showed that the ratio of AgNPs with carbopol (2 : 1), that is, F1, demonstrated maximum inhibition with a value of 75.16 ± 0.04. At the same time, the value differs with other ratios, namely, AgNPs with carbopol (1 : 1) F2 and AgNPs with carbopol (1 : 2) F3. Thus, it can be concluded that maximum concentration of apple extract revealed maximum percent inhibition.

### 3.4. Antimicrobial Activity of Silver Nanoparticulate Hydrogel

Apple extract loaded silver nanoparticles based hydrogels showed higher antimicrobial properties against the bacterial species of* E. coli *and* S. aureus*. Well-diffusion method was opted to evaluate the antibacterial activity. The inhibitory action of silver compounds may be attributed to the strong interaction of silver with thiol groups present in key respiratory enzymes in bacteria [31].

**Figure 6:**
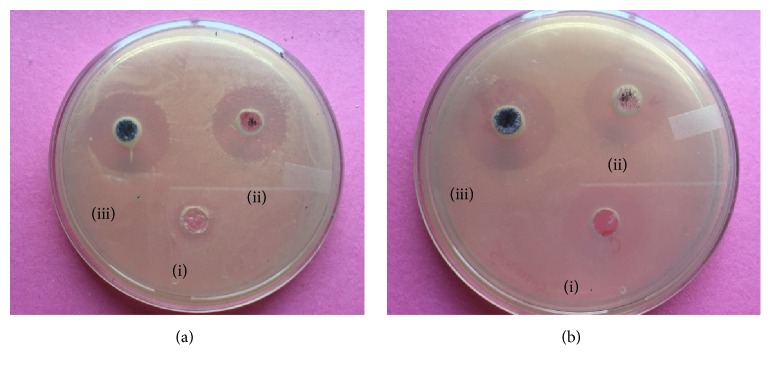
Antibacterial activity of silver nanoparticles (a) inhibition against* E. coli* and (b) inhibition against* S. aureus*. (i) Plain extract containing hydrogel, (ii) silver nanoparticles containing apple extract, and (iii) apple AgNPs loaded hydrogel.

The samples shows good zone of inhibition. Figures 6(a) and 6(b) show the zone of inhibition obtained against* E. coli *and* S. aureus,* respectively. A good inhibition was shown by silver nanoparticles based hydrogel contrary to plain hydrogel containing apple extract where no zone of inhibition was seen against* E. coli *as well as for* S. aureus*.

## 4. Conclusion

In the present research, green synthesis of apple extract loaded Ag-NPs was successfully achieved by treatment of silver nitrate with extract of* Malus domestica* (apple). This technique revealed that the apple extracts can be used as an effective stabilizing reducing and capping agent for the synthesis of AgNPs. This method of reduction utilized here is very simple, easy to perform, inexpensive, eco-friendly, and superior substitute to chemical synthesis. The finally fashioned AgNPs based hydrogels were highly stable formulations, showing strong antibacterial and antioxidant activity.

## Figures and Tables

**Table 1:** Formulation table for apple extract loaded silver nanoparticles based hydrogels.

S. number	Ingredients	Formulations		
		F1	F2	F3
(1)	Apple AgNPs	8 mL	4 mL	4 mL
(2)	Carbopol-934	4%	4%	8%
(3)	Glycerine	2 mL	2 mL	2 mL
(4)	Triethanolamine	1 mL	1 mL	1 mL
(5)	Distilled water	q.s. to 100 mL	q.s. to 100 mL	q.s. to 100 mL

**Table 2:** Evaluation parameters of apple extract loaded silver nanoparticles based hydrogels.

S. number	Parameters	F1	F2	F3
(1)	pH	6.11 ± 0.1	6.36 ± 0.15	6.66 ± 0.2
(2)	Viscosity	40000 cps	42000 cps	43000 cps
(3)	Spreadability	4.83 ± 0.3 g·cm/sec	5.56 ± 0.15 g·cm/sec	5.63 ± 0.15 g·cm/sec
(4)	Porosity	55.4%	61.2%	63.9%

**Table 3:** Release kinetics and permeation parameters for apple extract loaded silver nanoparticles based hydrogels.

Apple extract loaded AgNPs based hydrogel (F2)										
Zero-order		1st-order equation		Korsmeyer-Peppas equation		Higuchi equation		Flux (*µ*g/cm^2^/h)	Permeability coefficient (*P*) (cm/h)	Lag time (h)
*k*	*R* ^2^	*k*	*R* ^2^	*n*	*R* ^2^	*k*	*R* ^2^			
3.973	0.96	0.078	0.884	0.105	0.916	19.46	0.803	0.0124	0.315	1.763

**Table 4:** Antioxidant activity of apple extract silver nanoparticles loaded hydrogels (DPPH radical scavenging assay).

S. number	Formulations	Percent DPPH scavenging effect
(1)	F1	75.16% ± 0.04
(2)	F2	69.22% ± 0.02
(3)	F3	68.18% ± 0.03
